# Statin-Induced Rhabdomyolysis Due to Pharmacokinetic Changes From Biliary Obstruction in a Patient With Metastatic Prostate Cancer

**DOI:** 10.1177/2324709620947275

**Published:** 2020-08-05

**Authors:** Sai Prasad Desikan, Phillip Sobash, Andrew Fisher, Raman Desikan

**Affiliations:** 1 White River Health Systems, Batesville, AR, USA

**Keywords:** prostate cancer, rhabdomyolysis, obstructive jaundice, drug-drug interaction(s)

## Abstract

Statins work synergistically with androgen receptor blockers and androgen biosynthesis inhibitors, improving survival in patients with metastatic castration resistant prostate cancers (mCRPCs). Survival improvement is more pronounced for patients receiving androgen biosynthesis inhibitors compared with patients receiving androgen receptor blockers. A rare adverse interaction between simvastatin and abiraterone (Zytiga), an androgen biosynthesis inhibitor, was observed in a patient with mCRPC due to pharmacokinetic changes resulting from obstructive jaundice.

## Background

Prostate cancer is a disease of elderly men, wherein 6 out of 10 patients are over the age of 65. Hypercholesterolemia is another disease that affects this elderly age group and has been associated with increased aggressiveness (Gleason 8-10) of prostate cancer.^1^ Statin therapy is common in patients predisposed to prostate cancer and has been associated with decreased incidence and aggressiveness of prostate cancer.^2^ This association was stronger with increased total dose, longer periods of treatment, and hydrophobic statin agents.^3^ Metastatic castration resistant prostate cancers (mCRPCs) are treated with androgen receptor blockers and androgen biosynthesis inhibitors. Drug-drug interactions between statins and these agents have been studied and associated with improved cancer-specific survival and overall survival.^4^ Adverse drug interactions are rare between these 2 classes of medication. In this case report, we describe a unique adverse drug interaction between abiraterone and simvastatin in a patient with mCRPC.

## Case Presentation

A 66-year-old male was started on abiraterone acetate and prednisone for mCRPC 3 months prior to hospitalization. The patient had progressed on enzalutamide, which was started for progressive disease after surgical castration. The patient was on vacation after starting a regimen of abiraterone and low-dose prednisone. The patient’s family noticed that he was increasingly jaundiced and he returned home. On his drive back (approximately 2 days prior to presentation), the patient noticed gait disturbances and increasing proximal lower limb weakness while driving. He presented to his primary care physician with increasing jaundice, lower limb weakness, gait difficulty, light colored stool, and itching. He denied any bowel or bladder incontinence. Medications listed included the following: abiraterone, 1000 mg; prednisone, 5 mg daily (started 3 months prior); amlodipine, 5 mg daily; metoprolol, 25 mg BID (twice a day) for hypertension (started 3 years prior); and simvastatin, 20 mg daily for mild hyperlipidemia (started 40 mg 6-7 years prior, but patient reduced dose to 20 mg 3 years prior).

Physical evaluation was notable for deep icterus and lower limb weakness. Hip flexion and extension were noted to be 3/5 bilaterally. Knee extension and flexion were 4/5. Deep tendon reflexes and Babinski were normal. Sensation was intact in bilateral lower extremities.

## Investigations

### Laboratory Evaluation

Laboratory evaluation revealed elevated values: total bilirubin 10.6 mg/dL (0.2-1.3 mg/dL), direct bilirubin 4.8 mg/dL (0.0-0.3 mg/dL), aspartate aminotransferase 630 U/L (17-59 U/L), alanine aminotransferase 363 U/L, alkaline phosphatase 587 U/L (38-126 U/L), and creatinine kinase (CK) 6368 U/L (55-170 U/L). Creatinine was normal at 0.9 mg/dL. Additionally, CK peaked at 40 112 U/L (55-170 U/L) and decreased to 589 U/L on discharge 7 days later. After biliary stent placement on day 12, total bilirubin decreased to 6.70 mg/dL (Table 1).

**Table 1.** Creatinine Kinase Peaked on Day 3 of Admission but Declined With Response to Treatment as Noted on Day 7. AST, ALT, ALP, and Bilirubin (Day 21) Declined After Biliary Stent Placement on Day 12.

	Day 0	Day 3	Day 7	Day 21 after biliary stent
BUN (9-20 mg/dL)	25	18	18	9
Cr (0.8-1.5 mg/dL)	0.9	0.6	0.7	0.7
Total bilirubin (0.2-1.3 mg/dL)	10.60	10.20	11.10	6.70
AST (17-59 U/L)	630	1146	844	312
ALT (0-49 U/L)	363	572	671	197
ALP (38-126 U/L)	587	556	549	348
Creatinine kinase (55-170 U/L)	6368	40 112	589	40

Abbreviations: BUN, blood urea nitrogen; Cr, creatinine; AST, aspartate aminotransferase; ALT, alanine aminotransferase; ALP, alkaline phosphatase.

### Radiologic Evaluation

**Figure 1.**
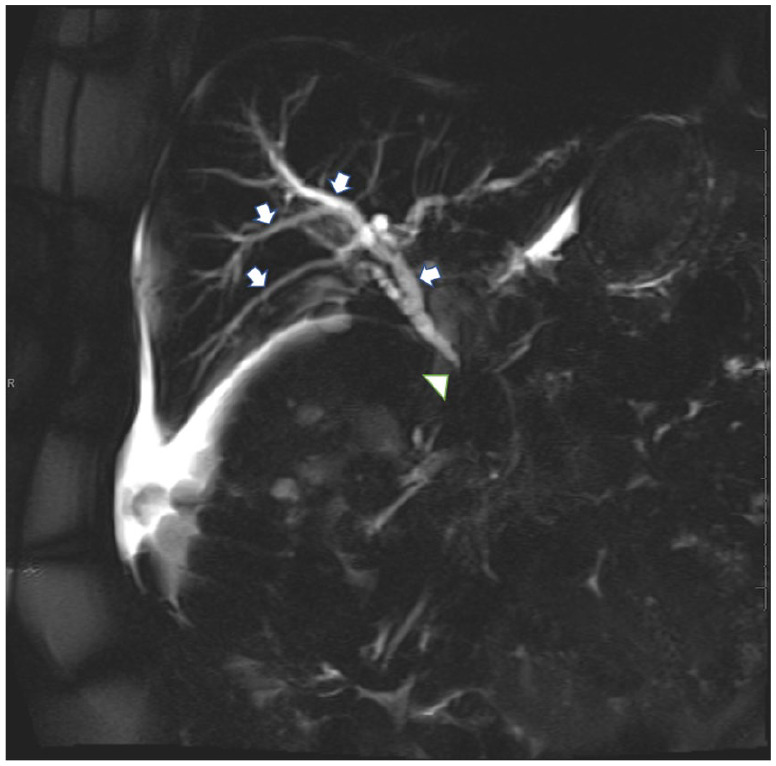
Single shot fast spin echo magnetic resonance image shows intrahepatic and extrahepatic bile duct dilatation (white arrows) with abrupt narrowing at the head of the pancreas (white arrowhead), compatible with biliary obstruction.

**Figure 2.**
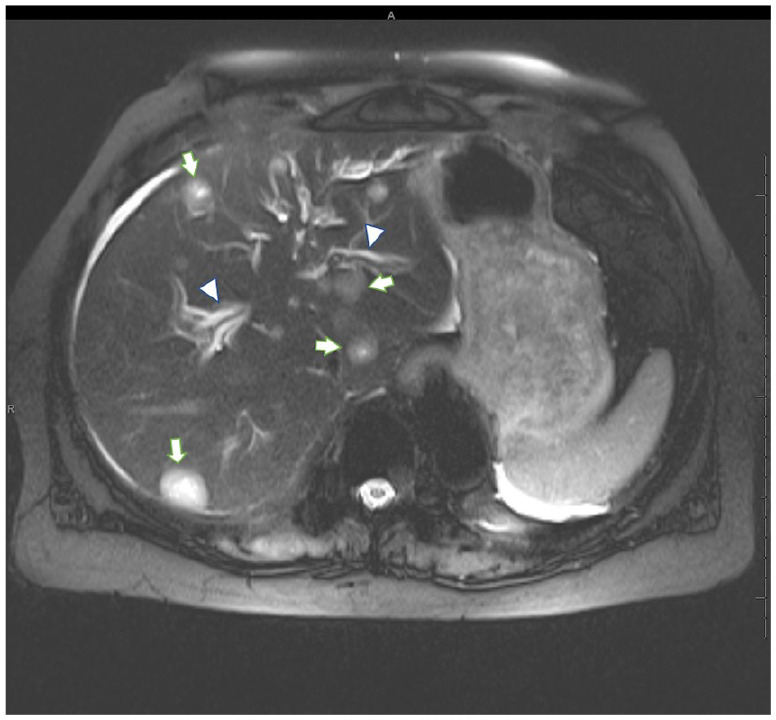
Axial T2 HASTE with fat saturation magnetic resonance image shows multiple intrahepatic T2 hyperintense lesions (white arrows), compatible with hepatic metastatic disease. Multiple dilated intrahepatic bile ducts (white arrowheads) noted as well.

Magnetic resonance imaging of the cervical, thoracic, and lumbar spine revealed bone metastasis without any evidence of cord compression. Hepatic ultrasound was significant for 2 hypoechoic liver lesions, which were concerning for metastasis, intrahepatic duct dilatation, and a borderline enlarged common bile duct measuring 6 to 7 mm. Magnetic resonance cholangiopancreatography showed intrahepatic and extrahepatic bile duct dilatation with abrupt tapering of the common bile duct at the head of the pancreas, which was concerning for extrinsic compression as well as lesions consistent with hepatic metastases (see Figures 1 and 2). Computed tomography–guided biopsy of one liver lesion was consistent with metastatic prostatic adenocarcinoma. Endoscopic retrograde cholangiopancreatography confirmed biliary stricture extending for 22 mm, and irregularity of the opacified pancreatic duct was also noted.

## Differential Diagnosis

Differential diagnoses on admission included spinal cord compression, jaundice related to liver metastasis, and abiraterone-induced liver injury.

## Treatment

Rhabdomyolysis was treated with aggressive intravenous fluids and discontinuation of simvastatin and abiraterone. CK level was 589 U/L on discharge. Weakness improved with physical therapy.

Biliary obstruction was relieved by biliary sphincterotomy, along with stenting of the common bile duct and pancreatic duct on day 12. Biopsy and cytology of the common bile duct were negative for malignant cells. In addition, the patient was initiated on taxotere for mCRPC on account of progression on enzalutamide and abiraterone.

## Outcome and Follow-up

The patient had increasing prostate-specific antigen levels on taxotere and was, hence, switched to cabazitaxel. Mutational profiling on tissue and peripheral blood was performed to identify any mutation that could be targeted.

## Discussion

Targeted therapy against specific mutated pathways is increasingly employed in the therapy of malignancies. Many targeted agents are small molecules, which can readily be formulated as oral therapy. While oral drugs offer ease of administration, they increase the odds of drug-drug interaction. Oral medication used to treat cancer may be substrates for, inducers of, or inhibitors of cytochrome P450 (CYP) enzymes involved in drug metabolism.^5^

Abiraterone is an oral CYP17 (working on 17-α hydroxylase and C17,20 lyase) inhibitor, which inhibits production of dehydroepiandrosterone (DHEA), a testosterone precursor.^6^ Simvastatin is a hydrophobic/lipophilic, HMG CoA reductase inhibitor.

While primarily used for hypercholesterolemia, simvastatin when employed with abiraterone improves overall survival, cancer-free survival, and improves response (prostate-specific antigen levels) in patients with mCRPC. This synergistic interaction is facilitated by a reduction in cholesterol (in and of itself a DHEA precursor), decreased DHEA transport across the cell membrane, and decreased steroid biosynthesis.^4^

Both simvastatin and abiraterone are primarily metabolized by CYP3A4 and excreted in bile. In addition to CYP3A4, CYP2D6 may be involved in simvastatin metabolism. Homozygous CYP2D6 mutations result in increased levels of simvastatin, thereby predisposing patients to statin-induced myopathy.^7^ Abiraterone is an inhibitor of CYP2D6 in addition to being a substrate for CYP3A4.^8^ Under normal conditions, CYP3A4 activity is adequate and both drugs can be used concomitantly.

Statins inhibit HMG-CoA reductase. HMG-CoA reductase converts HMG-CoA to mevalonate. Mevalonate is an essential precursor for production of isoprenoids, which are important for anchoring small GTPases.^5^ Inactivation of Rab GTPases, which are essential for intracellular vesicle trafficking, appears to be an important process in the development of rhabdomyolysis.^9^ Statin-induced rhabdomyolysis is dose dependent, especially when employed with agents that are also myotoxic or increase statin concentration. Pharmacokinetic changes induced by drug-drug interactions cause rhabdomyolysis in the majority of patients receiving statin therapy. Other risk factors for rhabdomyolysis include advanced age, frailty, pre-existent myopathy, hypothyroidism, as well as renal and liver function abnormalities.^10^

The patient presented with obstructive jaundice and lower limb weakness. Obstruction of the biliary tree leads to increased concentrations of both simvastatin and abiraterone in the patient. With increasing concentrations of abiraterone, abiraterone-induced CYP2D6 inhibition becomes more prominent. This inhibition in conjunction with competitive CYP3A4 inhibition (due to increased concentration of both drugs and metabolites) results in increased simvastatin levels and rhabdomyolysis.^11^ While there are a few case reports of abiraterone solely inducing rhabdomyolysis, our patient tolerated abiraterone and simvastatin until the onset of biliary obstruction, which precipitated the rhabdomyolysis. Amlodipine is also known to increase drug levels (area under the curve) of statins and results in dose-dependent myotoxicity when employed with statins.^12,13^ However, based on the dose and the fact that amlodipine is primarily renally excreted, it is suspected that this interaction did not contribute significantly to rhabdomyolysis.^14,15^

In the vast majority of patients, abiraterone and simvastatin do not demonstrate adverse drug-drug interaction and the coadministration of these agents improve survival and cancer-related mortality in mCRPC. This case highlights the importance of pharmacokinetic changes in precipitating drug-drug interactions. Rhabdomyolysis resulting from drug-drug interactions is a dynamic process that is affected by demographics, genetics, drug dosage, and pharmacokinetic changes. Careful attention to co-administered drugs is necessary to avoid drug toxicity when major changes in pharmacokinetics are anticipated.

## Learning Points/Take Home Messages

- Oral oncology medications are potential inducers/inhibitors of CYP enzymes.
- Simvastatin and abiraterone have a synergistic effect in patients with mCRPC.
- Pharmacokinetics are not static and can vary based on patient physiology.
